# The Role of *FLOWERING LOCUS C* Relatives in Cereals

**DOI:** 10.3389/fpls.2020.617340

**Published:** 2020-12-22

**Authors:** Alice Kennedy, Koen Geuten

**Affiliations:** Department of Biology, KU Leuven, Leuven, Belgium

**Keywords:** flowering time, cereals, FLOWERING LOCUS C, vernalisation, ambient temperature

## Abstract

*FLOWERING LOCUS C* (*FLC*) is one of the best characterized genes in plant research and is integral to vernalization-dependent flowering time regulation. Yet, despite the abundance of information on this gene and its relatives in *Arabidopsis thaliana*, the role *FLC* genes play in other species, in particular cereal crops and temperate grasses, remains elusive. This has been due in part to the comparative reduced availability of bioinformatic and mutant resources in cereals but also on the dominant effect in cereals of the *VERNALIZATION* (*VRN*) genes on the developmental process most associated with *FLC* in *Arabidopsis*. The strong effect of the *VRN* genes has led researchers to believe that the entire process of vernalization must have evolved separately in *Arabidopsis* and cereals. Yet, since the confirmation of the existence of *FLC*-like genes in monocots, new light has been shed on the roles these genes play in both vernalization and other mechanisms to fine tune development in response to specific environmental conditions. Comparisons of *FLC* gene function and their genetic and epigenetic regulation can now be made between *Arabidopsis* and cereals and how they overlap and diversify is coming into focus. With the advancement of genome editing techniques, further study on these genes is becoming increasingly easier, enabling us to investigate just how essential *FLC*-like genes are to modulating flowering time behavior in cereals.

## Introduction

*FLOWERING LOCUS C* (*FLC*) genes are a clade of MADS-box transcription factors in plants and are major regulators in many aspects of plant development. They are mostly associated with vernalization-regulated flowering but also have important roles in seed dormancy (Chen et al., 2014; Chen and Penfield, 2018), ambient temperature regulated development (Balasubramanian et al., 2006; Lee et al., 2013), germination (Chiang et al., 2009), as well as being associated with other processes like bud dormancy, circadian rhythm, water use efficiency, and indirect defense against herbivory (McKay et al., 2003; Edwards et al., 2006; Kumar et al., 2016; Mohammadin et al., 2017). In fact, there are over 500 FLC binding sites in the *Arabidopsis thaliana* (henceforth *Arabidopsis*) genome indicating that FLC is involved in much more than vernalization (Deng et al., 2011). In flowering time regulation, FLC acts as a repressor protein and acts mainly by repressing the activation of key floral promoting genes such as *FLOWERING LOCUS T* (*FT*) and *SUPPRESSOR OF OVEREXPRESSION OF CONSTANS 1* (*SOC1*; Searle et al., 2006; Deng et al., 2011).

**Figure 1:**
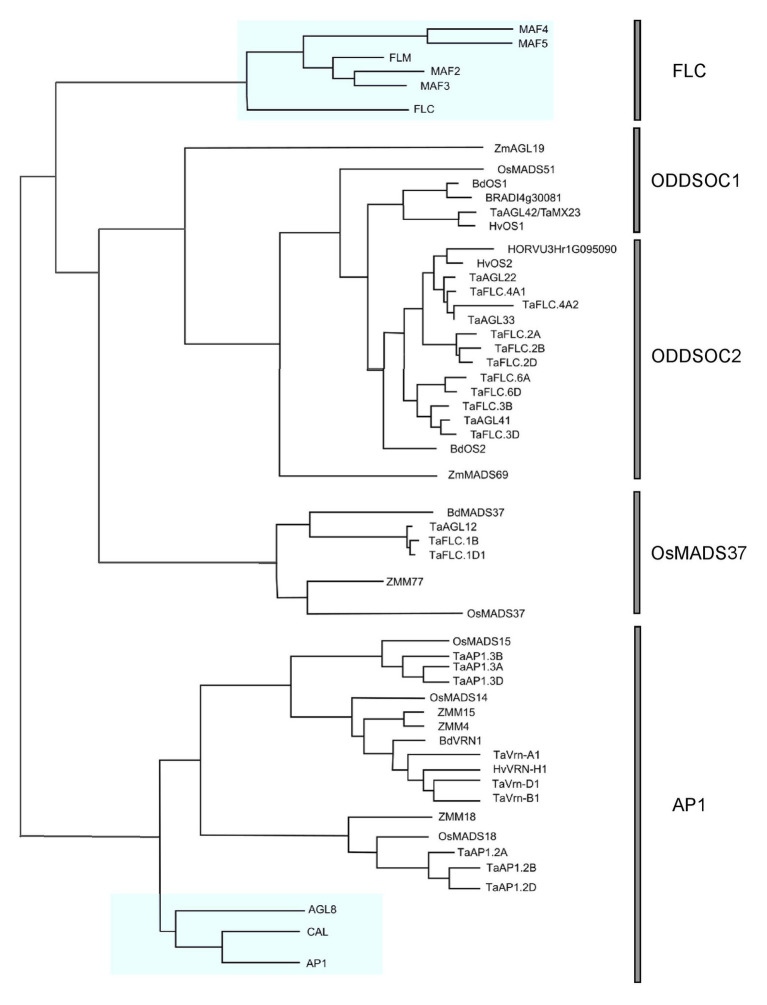
Phylogenetic relationships of all *FLOWERING LOCUS C* (*FLC*) genes annotated in cereals. A maximum likelihood phylogeny was generated using Geneious Pro v5.5.4 using MADS-box genes from eudicots and monocots. A reconstructed phylogeny containing only *FLC* and *AP1* genes was drawn using InkScape. *AP1* genes were used as an outgroup. *Arabidopsis* genes are highlighted in blue. A full version of the tree including all accession numbers can be found in Supplementary Figure S1.

The existence of *FLC*-like genes in cereals remained elusive for many years while the wealth of information on *Arabidopsis FLC* continued to accumulate. Many believed that *FLC* was restricted to eudicots and that monocot plants evolved separate mechanisms to regulate development and flowering time. However, a turning point came when it was concretely established through genome synteny analysis and phylogenetic reconstructions that *FLC* relatives did indeed exist in cereals (Ruelens et al., 2013). In this pivotal publication, it was shown that a clade of genes within monocots were phylogenetically related to the *FLC* genes of *Arabidopsis*. Within this monocot *FLC* clade, there are two subclades: the OsMADS51 and OsMADS37 subclades, so called after the representation of these rice genes within each clade. The OsMADS51 subclade is subsequently divided into two groups: the ODDSOC1-like and ODDSOC2-like groups. The name “ODDSOC” came from their weak similarity to the flowering time gene *SOC1* (Greenup et al., 2010). Members of the ODDSOC2 clade of genes are the most characterized out of all *FLC*-like genes in monocots thus far. The details of these relationships and their relationship to the *Arabidopsis FLC* genes can be seen in Figure 1. It must be noted that although the *Arabidopsis* and monocot *FLC* clades are related, it is likely that the ancestral gene function was partitioned differently within the groups. Therefore, direct comparisons of individual members across groups are not completely accurate.

Furthermore, *FLC*-like genes in cereals are Type II MADS-domain proteins despite having previously been annotated as Type I MADS-domain proteins, and have the typical MIKC protein structure (Zhao et al., 2006; Schilling et al., 2020).

Due to the advancement of genome sequencing technology, genetic mapping and genome editing methods, the nature of the function of *FLC*-like genes in cereals is coming into focus. Furthermore, avenues are now opening to further advance our knowledge on these genes which may reveal diversification of their function from their *Arabidopsis* homologs. This review aims to highlight the key findings over the last two decades of the role *FLC* relatives play in cereals and how progress made in biotechnology will further our understanding of the molecular control of plant development, perhaps leading us to utilize these genes as biotechnological tools for crop improvement.

## Evidence for Conserved Function: What do we Already Know?

### FLCs Are Involved in Vernalization in Grasses

In cereals, the main determinants of vernalization-regulated flowering are the *VERNALIZATION* (*VRN*) genes *VRN1*, *VRN2*, and *VRN3* (Yan et al., 2003, 2004, 2006). Generally, vernalization results in the upregulation of the floral promoter *VRN1* which downregulates the floral repressor *VRN2*, alleviating its repressive effect on the flowering promoter *VRN3*, an orthologue of *FT* (reviewed in Trevaskis et al., 2007; Distelfeld et al., 2009; Ream et al., 2012). VRN3 then positively regulates *VRN1* expression resulting in a positive feedback loop which induces flowering. This feedback loop in general determines the flowering habit of cereals with mutations in any of these proteins leading to altered spring or winter growth habit (Yan et al., 2004, 2006; Fu et al., 2005). Yet, variation in vernalization response can still remain in cultivars which have shared alleles of these genes (Rizza et al., 2016), opening up the potential for other genes to have functional significance in this process. The identification of *FLC*-like genes in cereal research remained elusive for many years leading to the conclusion that the vernalization pathway of *Arabidopsis* and cereals evolved completely separately (Yan et al., 2004; Winfield et al., 2009; Greenup et al., 2010). Incorporating the finding that an FLC clade exists in monocots, it appears more likely that the ancestral species of dicots and monocots contained both *FLC* and *AP1*/*VRN1*-like genes, and each group was differentially recruited during the evolution of vernalization responsiveness. As *AP1* retains a role in regulating flowering in *Arabidopsis*, so too do *FLC* homologs play a role in similar processes in cereals. This section aims to highlight these roles *FLC*-like genes play in the vernalization process and flowering time regulation in crop species.

*FLOWERING LOCUS C* homologs were described as being involved in vernalization in cereals almost 20 years ago where Trevaskis et al. (2003) described *TaMX23*, a MADS-box gene repressed by vernalization in winter wheats. *TaMX23* increases in abundance in early vegetative development and the effect of vernalization on *TaMX23* expression depended on whether the cultivar was a spring or winter variety (Trevaskis et al., 2003). *TaMX23* shares homology with both *TaODDSOC2* (*TaOS2*; also known as *TaAGL33*) and *TaAGL42* (Winfield et al., 2009). Although it is more similar in sequence to *TaAGL42*, its reported expression pattern reflects that of *TaOS2*.

*TaOS2* is the most described *FLC*-like gene in wheat so far. Like *FLC*, all three homeologs of *TaOS2* are downregulated by vernalization and repression is maintained 2 weeks post-vernalization (Winfield et al., 2009; Sharma et al., 2017; Appels et al., 2018). In winter varieties, *TaOS2* expression is initially high in leaf tissue and gradually declines throughout development as temperature decreases, yet its expression is constitutively low in spring lines, indicating that the function is cultivar-dependent and relevant to the flowering habit of these lines (Winfield et al., 2009; Sharma et al., 2017). Creating premature stop codons using CRISPR/Cas9 gene editing in the D-homeolog revealed an effect of this gene on flowering time, as mutants flower 3 days earlier than wild type (Appels et al., 2018). It is encouraging that a knockout of a single homeolog in hexaploid wheat reveals a phenotype. A 3-day alteration in flowering time is no mean feat in wheat breeding and can have great implications on yield in a region-specific manner. Validating this phenotype in the field will be enlightening to discover whether the phenotype is maintained and in which environments are the greatest effects found. It is also possible that functional redundancy is at play and multiple mutations in all homeologs of *TaOS2* might reveal more striking phenotypes to uncover the roles of these genes in flowering time regulation in wheat.

A second *FLC*-like gene, *TaAGL42* (or *TaODDSOC1*), has also been described as being regulated by vernalization in wheat (Winfield et al., 2009; Sharma et al., 2017); however, *TaAGL42* is upregulated in winter cultivars and is downregulated or stably expressed in spring varieties. Additionally, *TaAGL42* expression was shown to increase rapidly in response to a sudden drop in temperature in two winter varieties, suggesting that this gene could be involved in cold acclimation and tolerance in these lines (Winfield et al., 2009). In conclusion, although the gene is cold-regulated in a variety-specific manner, the function of *TaAGL42* remains unclear in wheat.

Relatives of these genes have also been described in barley, where their identification came about as a result of a desire to characterize new genes responsive to vernalization. Through the analysis of homologs of *TaMX23* (Trevaskis et al., 2003), two genes were identified: one sharing homology to *TaMX23* and another sharing homology with *TaOS2* (Trevaskis et al., 2003; Winfield et al., 2009; Greenup et al., 2010). The two homologs were named *HvODDSOC1* (*HvOS1*) and *HvODDSOC2* (*HvOS2*), respectively, due to their weak sequence similarity to *SOC1* in *Arabidopsis*. *HvOS1* expression increased in response to vernalization, consistent with its homolog in wheat (Winfield et al., 2009). *HvOS2* expression was repressed in response to vernalization in both the leaves and apices, and this repression was maintained post-vernalization (Greenup et al., 2010). The expression of *HvOS2* was also strongest in winter barley varieties pre-vernalization and was dramatically reduced upon exposure to prolonged cold, while expression remained low and constant in spring varieties. This highlights the importance of choice of cultivar when studying *FLC* homologs in cereals. Overexpressing *HvOS2* in the spring barley resulted in delayed flowering in these lines, strongly suggesting that *HvOS2* acts as a repressor of the floral transition. In contrast, no phenotype was observed for *HvOS2* knockdown lines created using RNA interference (RNAi); however, this is to be expected in a spring line where *HvOS2* is low naturally and vernalization is not required. In a separate study, differences in the rate of reproductive development under insufficient vernalization conditions was also explained by a difference in *HvOS2* expression levels between two winter varieties (Monteagudo et al., 2019), further supporting the idea of *HvOS2* as a vernalization-dependent regulator of the floral transition.

Aside from the crops themselves, research has been conducted on *FLC*-like genes in the model temperate grass *Brachypodium distachyon* (henceforth *Brachypodium*). In fact, *Brachypodium* was the organism chosen to first analyze the response of monocot *FLC* homologs to vernalization after they were first reported by Ruelens et al. (2013). Three homologs were reported in *Brachypodium*: *BdODDSOC1* (*BdOS1*), *BdODDSOC2* (*BdOS2*), and *BdMADS37*. *BdOS1* was shown to be upregulated by vernalization, like its homologs *TaAGL42* and *HvOS1* in wheat and barley, respectively. *BdOS2* expression is also consistent with its homologs in these species, as it is downregulated by vernalization (Ruelens et al., 2013; Sharma et al., 2017). There is also evidence to suggest that *BdOS2* pre-vernalization expression levels determine the vernalization requirement of individual *Brachypodium* accessions, with winter accessions having higher pre-vernalization expression levels of *BdOS2* (Sharma et al., 2017). Overexpression of *BdOS2* led to a delay in flowering time under vernalized conditions in the facultative accession Bd21–3, with the delay comparative to wild type plants which were not vernalized. This suggests that overexpression of *BdOS2* keeps Bd21–3 in a non-vernalized state. It was also reported that *BdOS2* knockdown *via* RNAi influenced the flowering time of Bd21–3; however, we and others have been unable to replicate these findings, calling these results into question. Attempts are currently being made to vigorously test the effect of low *BdOS2* expression on flowering time regulation in *Brachypodium*, with most striking phenotypes expected in winter accessions, and not facultative lines like Bd21–3.

The third *FLC* homolog, *BdMADS37*, is also downregulated by vernalization and exists in a separate clade to the ODDSOC genes (Figure 1). No other reports about members of this gene group have been published since first described by Ruelens et al. (2013); however, *BdMADS37* appeared as a potential candidate for a QTL explaining the differences in flowering time and vernalization requirement between spring and winter accessions under specific environmental conditions (Bettgenhaeuser et al., 2017). We have identified a fourth *FLC* homolog in *Brachypodium*, BRADI4g30081 (Figure 1), a paralog of *BdOS1* which appears to be a truncated duplication of *BdOS1*, and expression has been detected in response to cold in the microarray dataset of Priest et al. (2014).

### FLC and VRN1 Activities Are Entwined in Cereals

Much of what we have learned about *FLC*-like genes so far comes from basic research on flowering time regulation and vernalization in cereals. Therefore, many of these findings have been related to or are based on descriptions of the activities of VRN1. So far, a relationship between VRN1 and ODDSOC2 activity has been reported in wheat and its diploid relative *Triticum monococcum*, barley, and *Brachypodium*. In general, evidence exists to suggest that VRN1 is required to repress *ODDSOC2* post-vernalization to enable rapid flowering.

In *T. monococcum*, it was observed that *TmOS2* levels rose post-vernalization in mutant lines lacking functional *TmVRN1* while levels remained low in wild type lines. Analysis of *TmOS2* levels pre- and during vernalization showed that there was no difference in expression between wild type and mutant lines. It is only post-vernalization *TmOS2* levels that are affected by *TmVRN1* loss of function, suggesting that TmVRN1 is required to repress *TmOS2* post-vernalization but not to reduce its activity initially (Greenup et al., 2010).

Similar to *T. monococcum*, *HvOS2* expression is lowest in barley lines with dominant, active *VRN1* alleles, consistent with the hypothesis that VRN1 represses *OS2* in temperate cereals (Greenup et al., 2010). Supporting this hypothesis, it was subsequently reported that HvVRN1 binds to the *HvOS2* promoter in the spring variety Golden Promise (Deng et al., 2015). Several HvVRN1 binding sites have also been identified throughout the *HvOS2* locus (Monteagudo et al., 2019).

Likewise, there is an antagonistic relationship between *BdOS2* and *BdVRN1* expression patterns in *Brachypodium*. *BdOS2* expression is elevated in *BdVRN1* knockdown lines (Woods et al., 2016). As well as that, *BdOS2* expression is elevated in lines overexpressing *BdVRN2*, associated with low *BdVRN1* expression and delayed flowering (Woods et al., 2016). Interestingly, *BdOS2* expression patterns are not significantly influenced by overexpression of *BdVRN1* or knockdown of *BdVRN2*, indicating that the response of *BdOS2* to *BdVRN1* expression is qualitative and not dosage dependent.

This relationship between *VRN1* and *ODDSOC2* is conserved in hexaploid wheat, where *TaOS2* expression post-vernalization is linked to the nature of *VRN1* alleles found in a given cultivar (Dixon et al., 2019). This relationship has implications for flowering time behavior which will be discussed in more detail in the next section.

### ODDSOC2 and the Balancing Act of Vernalization and Ambient Temperature

Temperature is a key environmental signal which regulates many facets of plant development. Flowering time in both *Arabidopsis* and cereals is regulated by ambient temperature, with increasing temperatures generally resulting in earlier flowering times (McMaster and Wilhelm, 2003; Balasubramanian et al., 2006; Ejaz and von Korff, 2017; Dixon et al., 2018). Underlying this trait in *Arabidopsis* are the activities of FLC and its relative FLOWERING LOCUS M (FLM). FLM, like FLC, negatively regulates the floral transition, however, it is mostly involved in ambient temperature-dependent flowering (Balasubramanian et al., 2006; Lee et al., 2013; Posé et al., 2013). FLM functions as part of a repressor complex with another MADS-domain transcription factor, SHORT VEGETATIVE PHASE (SVP). This complex represses the activities of flowering promoters under cold temperatures to delay flowering and the stability of the complex and of the proteins themselves are affected by increasing temperature, reducing their repressive effects in warm conditions (Lee et al., 2013; Posé et al., 2013; Capovilla et al., 2017). Temperature-dependent alternative splicing of *FLM* is integral to this response, where the relative abundance of certain transcripts compared to others determines the flowering phenotype in response to temperature (Capovilla et al., 2017; Lutz et al., 2017).

High levels of *FLC* itself also results in thermal unresponsiveness, therefore, suggesting that FLC suppresses thermal induction of flowering (Balasubramanian et al., 2006). These findings could suggest that vernalization is the dominant process that must be realized to allow *Arabidopsis* to be receptive to temperature, likely to prevent precocious flowering in winter.

In cereals, the activities of *ODDSOC2* can also be linked to ambient-temperature regulated flowering. *ODDSOC2* has been shown to be responsive to ambient temperature in both wheat and barley, which both show earlier flowering phenotypes in response to increasing temperature (McMaster and Wilhelm, 2003; Ejaz and von Korff, 2017; Dixon et al., 2018). In wheat, however, it was shown that certain cultivars exhibited delayed flowering in response to increasing ambient temperature (Dixon et al., 2019). It was revealed that this trait arose from the incomplete vernalization of this cultivar, leading to the re-activation of floral repressors including *VRN2* and *TaOS2*. The increase in *TaOS2* expression was linked to the *VRN1* alleles found in this specific cultivar, which were unable to maintain repression of *TaOS2* after incomplete vernalization, explaining in part the delayed flowering phenotype (Dixon et al., 2019).

*HvOS2* was also shown to be responsive to ambient temperature in barley. *HvOS2* expression increases under high temperature conditions, particularly under short day photoperiods – conditions which result in the slowest development of the shoot apex (Hemming et al., 2012; Ejaz and von Korff, 2017). Like *TaOS2* in wheat, the response of *HvOS2* is influenced by the *VRN1* allele present in the variety analyzed, with lines with the winter *Hvvrn1* allele having higher *HvOS2* expression levels in response to high ambient temperature (Ejaz and von Korff, 2017). *Hvvrn1* itself is downregulated under high temperature conditions, highlighting further the negative correlation between *VRN1* and *OS2* expression in cereals. Plants overexpressing *HvOS2* also exhibited delayed reproductive development under both cool and high temperatures while lines with a RNAi-mediated knockdown of *HvOS2* underwent more rapid reproductive growth at higher temperatures compared to wild type plants (Hemming et al., 2012). This is reflective of phenotypes obtained when *FLM* expression is modified in *Arabidopsis* (Posé et al., 2013).

Taken together, this evidence suggests that *ODDSOC2* functions in winter cereal varieties to repress the reproductive transition under warm temperatures until the vernalization requirement is completely saturated. It can be speculated that this is an adaptation to prevent precocious flowering during the winter should a brief period of warmth occur. Interestingly, this ecologically significant process is linked to *FLC* gene activity across both *Arabidopsis* and cereals; however, unlike FLM, the mode of action of ODDSOC2 remains unknown. There is no evidence so far to suggest that *ODDSOC2* is alternatively spliced to influence this process. It is also unknown whether this increase in *ODDSOC2* expression occurs in varieties which can be induced to flower using warm temperatures and short-day conditions (Evans, 1987). Regardless, parallels can be drawn on the roles these *FLC* genes play in fine-tuning flowering time in a temperature-specific manner in both *Arabidopsis* and cereals.

**Figure 2:**
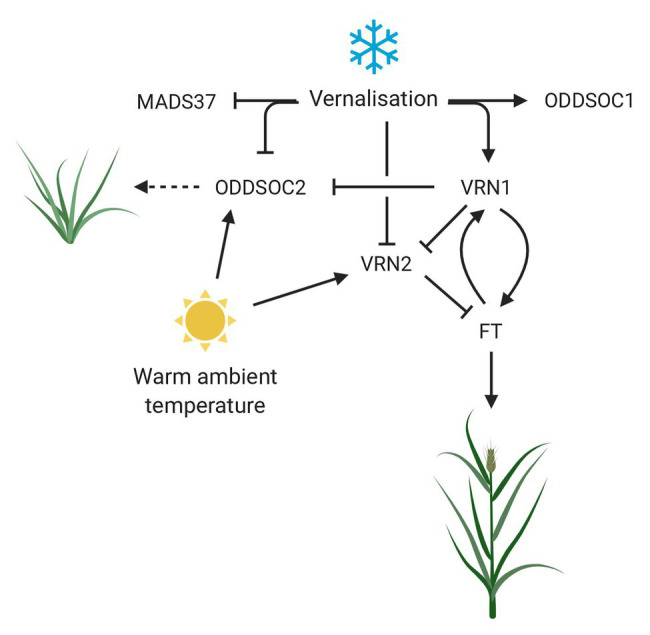
The role of *FLC* genes in the response to temperature in temperate cereals with a vernalization requirement. Vernalization represses the activities of floral repressors ODDSOC2 and VRN2, while promoting the upregulation of *VRN1*. VRN1 is required to maintain the repression of *ODDSOC2* and *VRN2* while promoting the flowering time gene *FT*, creating a positive feedback loop to lock in the floral transition. Warm ambient temperature during the vernalization process results in the upregulation of floral repressors to coordinate reproductive development with the most optimal environmental conditions. Dashed arrow implies function not confirmed. MADS37 and ODDSOC1 are included but their function is still unknown. Created with www.BioRender.com.

A summary of the roles *FLC* genes play in vernalization, ambient temperature, and their relationship to *VRN1* are outlined in Figure 2 and Table 1.

**Table 1:** Overview of *FLC* gene function in cereals.

Species	Gene	Observation	Citation
*Brachypodium distachyon*	*BdOS1*	Upregulated by vernalization	Ruelens et al. (2013)
	*BdOS2*	Downregulated by vernalization	Ruelens et al. (2013); Sharma et al. (2017)
		Negatively regulated by VRN1	Woods et al. (2016)
	*BdMADS37*	Downregulated by vernalization	Ruelens et al. (2013)
*Hordeum vulgare*	*HvOS1*	Induced by ABA and JA Role in seed development	Kapazoglou et al. (2012)
		Upregulated by vernalization	Greenup et al. (2010)
		Downregulated by high temperature	Hemming et al. (2012)
	*HvOS2*	Induced by JA Role in seed development	Kapazoglou et al. (2012)
		Downregulated by vernalization Negatively regulated by VRN1 Regulates cell elongation	Greenup et al. (2010)
		VRN1 binds to its promoter	Deng et al. (2015)
		Possible negative regulator of early reproductive development	Hemming et al. (2012); Monteagudo et al. (2019)
		Upregulated under high ambient temperature	Hemming et al. (2012); Ejaz and von Korff (2017)
*Oryza sativa*	*OsMADS51*	Short-day flowering promoter	Kim et al. (2007)
*Triticum aestivum*	*TaAGL33/TaOS2*	Downregulated by vernalization	Winfield et al. (2009); Sharma et al. (2017); Appels et al. (2018)
		Knockout of D-homeolog causes earlier flowering	Appels et al. (2018)
		Upregulated under high ambient temperature	Dixon et al. (2019)
	*TaAGL42/TaMX23/TaOS1*	Upregulated in response to cold	Winfield et al. (2009); Sharma et al. (2017)
		Gradual increase in expression throughout development	Trevaskis et al. (2003); Winfield et al. (2009)
*Triticum monococcum*	*TmODDSOC2*	Negatively regulated by VRN1	Greenup et al. (2010)
*Zea mays*	*ZmMADS69*	Flowering promoter	Liang et al. (2019)

### FLC Is Relevant Outside of Temperate Cereals

Rice and maize diverged from the Pooideae roughly 64 million years ago and their flowering times are regulated in different ways to the temperate cereals (Wang et al., 2015). Reflective of the tropical regions in which they evolved, these species have no vernalization requirement and flowering is promoted by short day photoperiods. Nonetheless, both rice and maize contain *FLC* homologs which have been shown to regulate flowering time. *OsMADS51*, a homolog of *ODDSOC1*, acts as a flowering promoter under short days in rice (subsp. Japonica; Kim et al., 2007). Knockout of this protein correlates with the downregulation of *AP1* and *FT* homologs, which may explain the mode of action of this protein. In maize, *ZmMADS69* acts as a flowering promoter under both long and short days, and is thought to have been a target of selection to expand the cultivation zone of maize (Liang et al., 2019). Therefore, despite paucity of information on *FLC*-like genes in cereals, it is clear that flowering time regulation is a fundamental feature of these genes across the cereals.

### Epigenetic Regulation of *FLC*-Like Genes in Cereals

The epigenetic regulation of *FLC* in *Arabidopsis* is well described and *FLC* can be considered a model gene for the study of epigenetics in general (Whittaker and Dean, 2017). *FLC* activity is regulated through the chromatin environment at the *FLC* locus and *via* RNA-mediated silencing mechanisms. Chromatin modification to promote *FLC* activity is regulated mainly through the actions of the FRIGIDA (FRI) complex (FRI-C). The FRI-C increases levels of active chromatin markers, such as H3K36me3 and H3K4me3, through the recruitment of chromatin modification proteins (Choi et al., 2011; Li et al., 2018). These markers are targeted during vernalization, where they are removed, and replaced with H3K9me3 and H3K27me3, resulting in a silenced chromatin state (Bastow et al., 2004; Finnegan and Dennis, 2007; Angel et al., 2011; Whittaker and Dean, 2017). The accumulation of chromatin silencing markers is mediated by the PHD-PRC2 complex (Plant Homeodomain-Polycomb Repression Complex 2; Wood et al., 2006; De Lucia et al., 2008).

Silencing of *FLC* is additionally associated with the action of long non-coding RNAs (lncRNAs) and components of the autonomous pathway (Sheldon et al., 2000; Ietswaart et al., 2012; Whittaker and Dean, 2017). RNA binding proteins of the autonomous pathway function to process *COOLAIR*, a set of lncRNAs transcribed antisense of *FLC* (Swiezewski et al., 2009; Hornyik et al., 2010; Whittaker and Dean, 2017). The significance of these lncRNAs in the regulation of vernalization remains controversial (Helliwell et al., 2011; Luo et al., 2019); however, much data have been gathered to indicate a functional if not essential role. The physical association of *COOLAIR* with *FLC* chromatin is associated with the reduction of H3K36me3 and H3K4me3, rendering the chromatin inactive (Csorba et al., 2014; Fang et al., 2020). *COOLAIR* is also induced by vernalization to assist with the inactivation of *FLC* (Swiezewski et al., 2009; Kim and Sung, 2017). The silencing of *FLC* is associated with two other lncRNAs, *COLDWRAP* and *COLDAIR*, which recruit the PHD-PRC2 complex to specific chromatin regions (Heo and Sung, 2011; Kim and Sung, 2017).

Due to the relatively recent discovery of *FLC*-like genes in cereals, no research has been done to test whether FRI or the FRI-C functions in cereal plants. Studies have shown that homologs of the various FRI-C components can be detected in monocots (Choi et al., 2011) and rice *FRI*-like genes form distinct clades with *Arabidopsis FRI*-like genes (Michaels et al., 2004). According to the plant genome database EnsemblPlants, 25 and 13 proteins have been annotated as FRI-like for *Triticum aestivum* (cv. Chinese Spring) and *Hordeum vulgare* (cv. Morex), respectively. Homologs can also be identified for *Brachypodium distachyon* (9), *Oryza sativa subsp. japonica* (12), *Sorghum bicolor* (10), *Triticum dicoccoides* (16), *Triticum turgidum* (15), and *Zea mays* (13). It is possible that these uncharacterized proteins may act as scaffold proteins similar to FRI but for other pathways and functions.

In *Arabidopsis*, *FLC* is silenced during vernalization *via* a series of histone modifications by the PHD-PRC2 complex (De Lucia et al., 2008). The utilization of this complex in plants as a method to regulate vernalization-dependent flowering is conserved across *Arabidopsis* and cereals. The major regulator of vernalization in cereals, *VRN1*, acts as a promoter of flowering, rather than a repressor like *FLC*. Before vernalization, H3K27me3 repressive marks are deposited at the *VRN1* locus in wheat and barley (Oliver et al., 2009; Diallo et al., 2012). During vernalization, H3K27me3 decreases while the active markers H3K4me3 and H3K36me3 increase (Oliver et al., 2009; Diallo et al., 2012). The same mode of epigenetic regulation of *VRN1* is conserved in *Brachypodium*, and is regulated by ENHANCER OF ZESTE-LIKE 1 (EZL1), a homolog of CURLY LEAF (CLF), and a methyltransferase in the PRC2 complex in *Arabidopsis* (Lomax et al., 2018). *VRN3/FT* is also regulated in the same way in wheat and *Brachypodium* (Oliver et al., 2009; Huan et al., 2018).

Recruitment of the PRC2 to epigenetically regulate the vernalization response evolved in both *Arabidopsis* and cereals. However, the nature of the chromatin modifiers deposited at *FLC* and *VRN1* is different due to their nature as a repressor and promoter, respectively. Therefore, this recruitment likely evolved after the independent evolution of the vernalization response pathway in monocots and dicots. Yet, some evidence exists to suggest that *FLC*-like genes are also regulated by the PRC2 in a similar manner to *FLC* in *Arabidopsis*. To analyze the effect of vernalization on histone modifications at *HvOS2* in barley, H3K27me3 marks were analyzed at the presumed transcriptional start site of *HvOS2* in plants with or without 7 weeks of vernalization (Greenup et al., 2010). There was no significant difference in H3K27 trimethylation at this region indicating that perhaps repression of *HvOS2* post-vernalization is regulated in a different way to *FLC*. The region tested by Greenup et al. begins ~100 bp upstream of the transcriptional start site (TSS). Although H3K27me3 deposits increase during vernalization at the TSS of *FLC* in *Arabidopsis*, the greatest increase is at the exon 1/intron 1 junction termed the “nucleation region” (Bastow et al., 2004; Finnegan and Dennis, 2007; Yuan et al., 2016). On return to warmth, H3K27me3 spreads from the nucleation region across the *FLC* locus. Future experiments targeting other regions within the *HvOS2* locus could reveal more similarities in the epigenetic regulation of both *FLC* and *HvOS2*. Regulation *via* other markers such as H3K9me3 or H3 acetylation levels could be investigated, as these markers are also involved in *FLC* regulation.

In contrast to barley, *BdOS2* in *Brachypodium* showed high levels of H3K27me3 after vernalization in both spring and winter accessions (Sharma et al., 2017). H3K27me3 was enriched at the *BdOS2* locus after vernalization for Bd21 and BdTR3C – spring and winter accessions, respectively, and the enrichment was maintained 1-week post-vernalization. For the winter allele of *BdOS2* in BdTR3C, H3K27me3 can be found spanning the entire locus post-vernalization. The extensive methylation marks of H3K27 in the locus of BdTR3C compared to Bd21 may explain the mechanism as to how *BdOS2* is stably repressed in the winter but not spring accession. It is possible that in strong winter varieties, *FLC* genes have evolved increasingly stringent or more complex methods of silencing to ensure flowering time is synchronized most optimally with the environment (Shindo et al., 2006; Hepworth et al., 2020). Analysis of winter varieties of *FLC* homologs in other cereal crops may reveal that the epigenetic regulation of *FLC*-like genes is more conserved than currently realized.

*Brachypodium* has also been shown to encode lncRNAs, similar to *COOLAIR*, which target *FLC*-like genes for downregulation during vernalization (Jiao et al., 2019). Two high confidence lncRNAs were detected for *BdOS2*, while one lncRNA could be detected for *BdOS1*. These lncRNAs were termed *BdCOOLAIR1* and *BdCOOLAIR2* for *BdOS1* and *BdOS2*, respectively, as although they are not homologous to the *AtCOOLAIR* sequence, their position relative to their sense counterparts is similar. Expression of these lncRNAs is induced by vernalization, and their induction is significantly higher in a winter accession compared to a facultative accession, while their expression is absent in a spring accession. Knockdown of *BdCOOLAIR2 via* RNAi also affects the rate of silencing of *BdOS2* in BdTR3C, though it is not essential for the complete silencing of *BdOS2* (Jiao et al., 2019). This information suggests that lncRNAs complement the mechanisms which silence *FLC*-like genes in grasses, in a similar fashion to *FLC* regulation in *Arabidopsis*, although this mode of regulation is accession dependent. In addition, lncRNAs have been annotated for *FLC* genes in 6 other grass species, including wheat, although these still need to be experimentally verified.

## Diversification of FLC Function in Crops

Although thoroughly studied for its involvement in vernalization-dependent regulation of flowering time, FLC function is implicated in many other aspects of plant growth and development. Analysis of expression levels of *FLC*-like genes in cereals during development and under various experimental treatments also suggests that homologs of *FLC* play diverse roles in cereal physiology. For example, in the early gene expression experiments of Zhao et al. (2006), it was shown that at least one of the genes analyzed is expressed at, at least, one of the various life stages and in at least one of the various tissue types throughout wheat development. Expression can be detected from initial embryo imbibition to seed development post-anthesis while other genes are predominantly expressed in roots.

Curiously, in the dataset of both Zhao et al. (2006) and Schilling et al. (2020), *TaAGL41* could not be detected at significant levels but could be detected by Sharma et al. (2017) for several cultivars. Further analysis using the Wheat Expression Browser (Ramírez-González et al., 2018) indicates that *TaAGL41* is indeed expressed throughout development but the extent of its expression is cultivar-specific. This could suggest a role for this gene in fine tuning development in a cultivar-specific manner. The Wheat Expression Browser highlights that *TaAGL41* is downregulated by cold in the spring cultivar Manitou (Li et al., 2015), yet the main stimulus which affected *TaAGL41* across the dataset was infection by the wheat yellow rust pathogen *Puccinia striiformis f*. sp. *Tritici* (Dobon et al., 2016). Expression of two other high confidence *FLC* homologs in wheat, *TaFLC.4A1* and *TaFLC.4A2*, could not be detected at significant levels in the developmental time course analyzed by Schilling et al. (2020), and their expression does not change considerably across the different varieties available on the Wheat Expression Browser. Rather, these genes appear to be mainly influenced by drought stress (Liu et al., 2015; Ramírez-González et al., 2018). The relationship between flowering time and stress adaptation is complex and the molecular mechanisms determining this relationship are still not fully understood; however, a link between flowering time regulators and stress is found in plants (reviewed in Kazan and Lyons, 2016). It is possible that *FLC*-like genes not only play roles regulating development but also that their function has diversified to fine tune other developmental and growth processes. This reflects findings in the Brassicaceae that although the core function of *FLC* across species is the regulation of flowering time, different members of the *FLC* clade have been recruited for species-specific roles typically within stress response pathways (Mateos et al., 2017). Further investigation into these expression patterns as well as generation of knockout mutants may reveal a novel role for these genes in stress response pathways in cereals.

Additionally, *HvOS2* has been shown to negatively influence cell length and, therefore, leaf, internode, and spike length (Greenup et al., 2010). The data suggest that *HvOS2* downregulation by vernalization allows the process of stem elongation and bolting as secondary regulation of the reproductive process.

As well as being expressed during seed development stages in wheat, *FLC*-like genes have been implicated in seed development in barley. *HvOS1* and *HvOS2* are differentially expressed in cultivars of different seed size and at different stages of seed development (Kapazoglou et al., 2012). Analysis of their expression patterns revealed that *HvOS1* expression is induced more substantially in early seed development in cultivars with large seeds, while *HvOS2* levels are significantly higher in later developmental stages in cultivars with small seeds. This pattern could suggest an association between the expression of *FLC* genes and seed size in barley and that each gene is important for different stages of development – either endosperm cellularization or seed maturation. Additionally, both genes contained the endosperm-specific element GCN4 in their promoters, along with elements for responses to abscisic acid, an important phytohormone for seed maturation as well as abiotic stress (Takaiwa et al., 1996; Finkelstein et al., 2002; Kapazoglou et al., 2012). Taken together, these data implicate a role of *FLC* homologs in seed development and suggest that perhaps there is an association between them and seed size. An association study including more cultivars with a variety of seed sizes could be undertaken to fully determine whether *FLC*-like genes regulate this important agronomic trait.

Outside of the temperate cereals, *ZmAGL19*, an *FLC* homolog in maize, is targeted by OPAQUE11, a central regulator of endosperm development and nutrient metabolism (Feng et al., 2018). *OPAQUE11* is specifically expressed in the endosperm and positively regulates *ZmAGL19* expression, suggesting that *ZmAGL19* might be part of the seed development regulation process in maize.

## Future Directions

Much has been learned about the roles *FLC* genes play in cereals, mostly indirectly through the study of flowering time in these species. The scientific community is now able to study *FLC* genes further due to the dramatically improved genetic resources available. Reference genome assemblies are now available for several hexaploid wheat cultivars, as well as tetraploid wheat, diploid progenitor species, and 2- and 6-row barley (Ling et al., 2013; Fox et al., 2014; Luo et al., 2017; Mascher et al., 2017; Appels et al., 2018; Maccaferri et al., 2019). Genes are also annotated to include SNP variations which can be easily identified using the online platform EnsemblPlants. Identifying homologs and SNP-variants across cultivars has never been easier for researchers without bioinformatics training. Access to tools such as these will increase the pace at which genes are identified and studied, increasing the potential to finally characterize the once enigmatic *FLC* gene family.

Additionally, populations of mutant plants have been created for widespread use in both hexaploid and tetraploid wheat and barley (Krasileva et al., 2017; Schreiber et al., 2019). TILLING lines containing homeolog-specific mutations in genes of interest can be ordered and crossed, creating specific combinations to study gene function and redundancy. In a more targeted approach, protocols for wheat transformation and mutation *via* virus-induced gene silencing and CRISPR/Cas9 are available (see wheat-training.com for resources). In combination with speed breeding, it is possible to fully characterize the effect of mutations in both model and crop plants in considerably less time (Watson et al., 2018). At this moment in time, comparable resources to the model plant *Arabidopsis* from which most of our information on *FLC* genes comes from are available. This review also highlights how relevant *Brachypodium* is as a model for basic and translational research for temperate cereals and that research using this small grass will continue to be a valuable option to study *FLC* genes. It is possible within the next few years that we will see a greater increase in *FLC*-related knowledge outside of *Arabidopsis*. The availability of these resources provides hope that much more knowledge can be gained on *FLC* function in cereals in years to come.

## Author Contributions

AK prepared the outline and wrote the manuscript. KG contributed to discussions and critical revision of the manuscript. All authors contributed to the article and approved the submitted version.

### Conflict of Interest

The authors declare that the research was conducted in the absence of any commercial or financial relationships that could be construed as a potential conflict of interest.
