# Supplementary figures and images for: The Role of FLOWERING LOCUS C Relatives in Cereals
Source: Front Plant Sci. 2020 Dec 22;11:617340. doi: 10.3389/fpls.2020.617340 (PMC7783157; doi:10.3389/fpls.2020.617340)

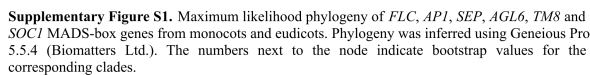

Supplement: Supplementary file 1 [file Data_Sheet_1.pdf]
